# Effect of *Helicobacter pylori* Eradication on TLR2 and TLR4 Expression in Patients with Gastric Lesions

**DOI:** 10.1155/2015/481972

**Published:** 2015-03-22

**Authors:** Aline Cristina Targa Cadamuro, Ana Flávia Teixeira Rossi, Joice Matos Biselli-Périco, Patrícia Fucuta Pereira, Edla Polsinelli Bedin Mascarin Do Vale, Ricardo Acayaba, Kátia Ramos Moreira Leite, Eny Maria Goloni-Bertollo, Ana Elizabete Silva

**Affiliations:** ^1^Department of Biology, São Paulo State University (UNESP), São José do Rio Preto Campus, Rua Cristóvão Colombo 2265, 15054-000 São José do Rio Preto, SP, Brazil; ^2^Base Hospital, Ambulatory of Gastrohepatology, Avenida Brigadeiro Faria Lima 5544, 15090-000 São José do Rio Preto, SP, Brazil; ^3^João Paulo II Hospital, Rua Doutor Eduardo Nielsem 960, 15030-070 São José do Rio Preto, SP, Brazil; ^4^School of Medicine, São Paulo University (USP), Avenida Dr. Arnaldo 455, 01246-903 São Paulo, SP, Brazil; ^5^School of Medicine, FAMERP, Avenida Brigadeiro Faria Lima 5416, 15090-000 São José do Rio Preto, SP, Brazil

## Abstract

*Objective*. *Helicobacter pylori* (Hp) is recognized by TLR4 and TLR2 receptors, which trigger the activation of genes involved in the host immune response. Thus, we evaluated the effect of eradication therapy on TLR2 and TLR4 mRNA and protein expression in *H. pylori*-infected chronic gastritis patients (CG-Hp+) and 3 months after treatment. *Methods*. A total of 37 patients CG-Hp+ were evaluated. The relative quantification (RQ) of mRNA was assessed by TaqMan assay and protein expression by immunohistochemistry. *Results*. Before treatment both *TLR2* and *TLR4* mRNA in CG-Hp+ patients were slightly increased (*TLR2* = 1.32; *TLR4* = 1.26) in relation to Hp-negative normal gastric mucosa (*P* ≤ 0.05). After successful eradication therapy no significant change was observed (*TLR2* = 1.47; *TLR4* = 1.53; *P* > 0.05). In addition, the *cagA* and *vacA* bacterial genotypes did not influence the gene expression levels, and we observed a positive correlation between the RQ values of *TLR2* and *TLR4*, both before and after treatment. Immunoexpression of the TLR2 and TLR4 proteins confirmed the gene expression results. *Conclusion*. In conclusion, the expression of both *TLR2* and *TLR4* is increased in CG-Hp+ patients regardless of *cagA* and *vacA* status and this expression pattern is not significantly changed after eradication of bacteria, at least for the short period of time evaluated.

## 1. Introduction

The* Helicobacter pylori* (*H. pylori*) bacterium is responsible for 5.5% of all infection-associated cancers [1] and is the major cause of gastric cancer in consequence of chronic inflammation. Persistent gastric mucosa inflammation results in chronic gastritis and progresses through a multistep process to gastric atrophy, intestinal metaplasia, dysplasia, and finally carcinoma [2]. The clinical consequences of* H. pylori* infection are determined by bacteria virulence genes as well as by host genetic factors such as immune response genes, besides environmental factors [3–5]. Among the bacterial products, the CagA (cytotoxin-associated gene A) and VacA (vacuolating cytotoxin) proteins are the major virulence factors related to the severity of gastric lesions and cell responses [6, 7].

The gastric epithelium cells provide the first point of contact for* H. pylori* adhesion through interaction with Toll-like receptors (TLRs), responding to the infection by activating various signaling pathways [8]. TLRs are key regulators of both innate and adaptive immune responses, recognizing several microbial products, such as lipoproteins, peptidoglycans, and lipopolysaccharides (LPS) [9]. The LPS of* H. pylori* is recognized mainly not only by TLR4 [10], but also by TLR2, which recognizes other forms that are structurally different from those recognized by TLR4 [11]. Both TLR2 and TLR4 are activated, after the bacteria recognition, in cooperation with the adapter molecule MyD88, triggering the mitogen-activating protein kinase (MAPK) signaling pathway. At this point, there is a subsequent activation of the transcription factor NF-*κ*B, which leads to the rapid expression of inducible nitric oxide synthase (iNOS) and proinflammatory cytokines, chemokines and their receptors, and interleukins [12, 13]. When these factors are stimulated, they initiate a marked inflammatory response of the mucosa, characterized as chronically active gastritis, and may acquire oncogenic potential [14, 15].

So far, the strategy for prevention of* H. pylori*-associated gastric cancer has been the eradication of these bacteria, regarded as a first-line therapy to reverse the preneoplastic lesions and prevent malignant progression [16]. However, treatment is not adopted for asymptomatic carriers in developing countries, due to its high cost [17].* H. pylori* is susceptible to most antibiotics, although resistance has been common, and triple or quadruple therapy consisting of two antibiotics, a proton pump inhibitor, and bismuth has lately been used to eradicate the bacteria [18]. Unfortunately, the eradication is not always successful, mainly due to chemoresistance [19]. Studies to evaluate changes in expression levels of genes involved in the recognition of the bacteria and the immune response of the host in patients infected by* H. pylori* are scarce, both before and after eradication treatment. Moreover, there are no reports about the expression of TLR2 and TLR4 in gastric lesions before and after bacterial clearance. Therefore, the main goal of the present study was to evaluate, for the first time, the mRNA and protein expression levels of TLR2 and TLR4 in* H. pylori-*infected chronic gastritis patients and the occurrence of changes in the expression levels of these receptors after successful* H. pylori* eradication therapy.

## 2. Materials and Methods

### 2.1. Patients

At first, 59 patients scheduled for upper endoscopy with positive histological and molecular diagnosis for* H. pylori* and not yet submitted to eradication therapy were enrolled prospectively between May 2010 and December 2012 from the Gastro-Hepatology Outpatient Clinic at the Base Hospital and the João Paulo II Hospital, both at São José do Rio Preto, SP, Brazil.

From each patient, gastric biopsies of the antrum region were collected for histological analyses and molecular and immunohistochemical studies. None of the individuals had taken any antibiotics, nonsteroidal anti-inflammatory drugs, or corticosteroids during the two months prior to endoscopy, nor did they take proton pump inhibitors or H_2_ antagonists in the 15 days preceding the procedure. Patients with gastric cancer and infectious diseases were excluded from this study. Gastric biopsy specimens were examined histologically by a specialized pathologist for the presence of the bacteria and histopathologically classified as superficial chronic gastritis (*n* = 45; mean age 44 years; 19 females and 17 males), atrophic gastritis (*n* = 8; mean age 50 years; 3 females and 5 males), and atrophic gastritis with intestinal metaplasia (*n* = 6; mean age 50 years; 4 females and 2 males), according to the Sydney system [20], constituting the so-called CG-Hp+ group. Of the 59 CG-Hp+ patients, only 37 (63%) concluded the treatment and were called completed treatment group, and 23/37 (62%) of them had the bacteria eradicated, as evidenced by concordant histological and molecular* H. pylori-*negative diagnosis. However, 14/37 (38%) remain infected showing histological or molecular* H. pylori-*positive diagnosis (Table 1). Four gastric biopsy specimens presented histologically normal* H. pylori*-negative gastric mucosa (normal Hp- group) and were used as control (mean age 35.6 years; 3 females and 1 male). Epidemiological data of patients and controls were collected using a standard interviewer-administered questionnaire, containing questions about smoking habits, alcohol intake, previous or ongoing treatment, use of medications, previous surgeries, and family history of cancer.

The CG-Hp+ group was submitted to standard triple therapy consisting of amoxicillin (1 g), clarithromycin (500 mg), and omeprazole (20 mg), all given twice daily for seven days. Three months after treatment, the individuals underwent another endoscopy for collection of gastric biopsies of the antrum region. Immediately after collection, the biopsy specimens were placed into RNA Later solution (*Applied Biosystems*) and stored at −20°C until nucleic acid extraction.

The study protocol was approved by the local Research Ethics Committee (CEP/IBILCE/UNESP number 030/10), and written informed consent was obtained from all participating individuals.

### 2.2. Molecular Diagnosis for* H. pylori* and* cagA* and* vacA* Genotypes

DNA/RNA extraction from the gastric biopsies was performed according to the protocol accompanying the reagent Trizol (*Invitrogen*) and the concentrations were determined in a NanoDrop ND1000 spectrophotometer (*Thermo Scientific*). Firstly, multiplex PCR was performed, using 100 ng of DNA in a final volume of 25 *μ*L containing specific primers for* H. pylori* genes such as* UreA *and* tsaA*, besides the constitutive human* CYP1A1* gene, according to our protocol which was described in previous study [21]. Molecular diagnosis was considered positive when at least one gene (*UreA* or* tsaA*) had been amplified. The* H. pylori*-positive samples were also subjected to PCR for investigation of polymorphisms in the sm regions of the gene* vacA* as previously described [22]. Primers amplify s1 fragment of 176 bp or s2 fragment of 203 bp, while primers for “m” alleles amplify m1 fragment of 400 bp or m2 fragment of 475 bp. Positive and negative controls were used in all experiments.

### 2.3. TaqMan Quantitative Real Time PCR (qPCR) for* TLR2* and* TLR4* mRNA

Reverse transcription (RT) of total RNA was performed using a High Capacity cDNA Archive Kit (*Applied Biosystems*), in a total volume of 25 *μ*L, according to the manufacturer's protocol. Then, qPCR was carried out in a* StepOnePlus Real Time PCR System  2.2.2 *(*Applied Biosystems*), using specific TaqMan probes for target genes* TLR2* (assay ID Hs00610101_m1,* Applied Biosystems*) and* TLR4 *(assay ID Hs01060206_m1,* Applied Biosystems*) and two reference genes,* ACTB* (part number: 4352935E,* Applied Biosystems*) and* GAPDH* (*glyceraldehyde 3-phosphate dehydrogenase*) (part number: 4352934E,* Applied Biosystems*), used as endogenous controls according to the manufacturer's instructions. All reactions were performed in triplicate in a final volume of 20 *μ*L, using 100 ng/*μ*L cDNA and a blank to ensure the absence of contamination. Relative quantification (RQ) of* TLR2 *and* TLR4 *mRNA was obtained according to the model proposed by Livak and Schmittgen [23] and normalized to the* ACTB *and* GAPDH *reference genes and a pool of normal Hp- samples.

### 2.4. Immunohistochemical Assay for TLR2 and TLR4 Proteins

Immunohistochemical analysis was performed in 14 samples from the CG-Hp+ group before and after bacteria eradication and four samples from the normal Hp- group. Consecutive 4 *μ*m thick sections were cut from each trimmed paraffin block. Deparaffinized tissue slides were then submitted to antigen retrieval, using a high-temperature antigen-unmasking technique. The sections were incubated with specific primary antibodies: rabbit polyclonal antibody anti-TLR2 (06-1119, 1 : 50 dilution;* Millipore*) and mouse monoclonal anti-TLR4 (76B357.1, 1 : 200 dilution;* Abcam*). Then the slides were incubated with biotinylated secondary antibody (Picture Max Polymer Detection Kit,* Invitrogen*) for 30 minutes, following the manufacturer's protocol. Immunostaining was done with 3,3′-diaminobenzidine tetrahydrochloride (DAB) containing 0.005% H_2_O_2_, counterstained with hematoxylin. Placenta mucosa and appendix tissue were used, respectively, as positive controls for the TLR2 and TLR4 proteins. The immunostaining was evaluated in the cytoplasm by densitometric analysis with an arbitrary scale going from 0 to 255, performed with Axio Vision software under a Zeiss-Axioskop II light microscope. Sixty equally distributed points were scored in each one of the regions, and the results were expressed as mean ± SE.

### 2.5. Statistical Analysis

Data analysis was performed using the computer software GraphPad Prism 5 version 5.01. The distribution of continuous data was evaluated using the D'Agostino and Pearson omnibus normality test or Shapiro-Wilk normality test. Data are presented as median and range, as mean ± standard deviation (SD), or as frequencies, according to the data distribution. Student's* t*-test for paired and unpaired data or correspondent nonparametric tests, such as the Mann-Whitney test and the Wilcoxon signed rank test, were used for comparisons between groups. To evaluate the association between relative gene expression and risk factors such as age, gender, smoking, drinking, and bacterial virulence genotypes, the Mann-Whitney test was performed. The correlation between* TLR2 *and* TLR4* mRNA expression before and after eradication therapy was analyzed using Spearman's Correlation. For protein expression, the means obtained from the densitometry analysis were compared before and after treatment and with the normal Hp- group using ANOVA followed by the Bonferroni test. The level of significance was set at *P* ≤ 0.05.

## 3. Results

### 3.1. The Relative Expression of* TLR2* and* TLR4* mRNA Is Not Changed after Successful Eradication Therapy

**Figure 1:**
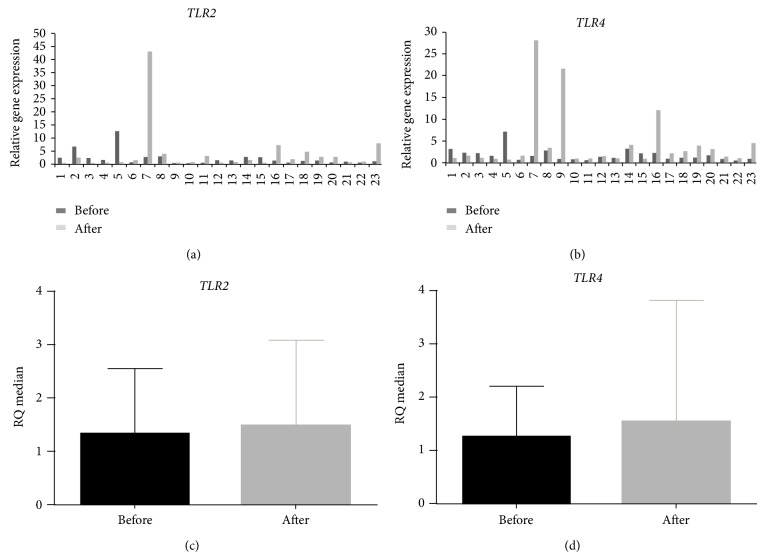
Relative expression levels of* TLR2* and* TLR4* RNAm in the eradicated patients group with chronic gastritis before and after* H. pylori *treatment. Relative quantification (RQ) of the mRNA expression levels of (a)* TLR2* and (b)* TLR4 *per individual evaluated; RQ median of (c)* TLR2* and (d)* TLR4* mRNA before and after* H. pylori* eradication. Data are presented as median and range for experiments performed in triplicate. Statistical significance was determined using Wilcoxon's signed rank test.

Table 2 shows the data regarding the relative expression levels of* TLR2* and* TLR4 *mRNA of 37 CG-Hp+ patients who concluded the treatment (completed treatment group), 23 CG-Hp+ patients in which the bacteria were eradicated, allowing paired analysis before and after eradication therapy, and 14 CG-Hp+ patients in which the bacteria were noneradicated. The relative expression levels of* TLR2* and* TLR4* mRNA after normalization with the* ACTB* and* GAPDH* reference genes and comparison with normal mucosa* H. pylori*-negative in all groups, either before or after treatment, were increased significantly (*P* < 0.05). Considering all patients that completed the treatment, no significant change was found after treatment in the relative expression levels of either* TLR2* or* TLR4* mRNA (*TLR2* = 1.55 and* TLR4* = 1.64) in comparison to the same cases before the treatment (*TLR2* = 1.31 and* TLR4* = 1.45). In the group that eradicated the bacteria, heterogeneity of relative expression levels for both* TLR2* and* TLR4* mRNAs can be observed before and after the treatment (Figures 1(a) and 1(b)). However no significant differences were observed for both genes comparing the expression levels in this group before and after treatment (*P* = 0.533 and *P* = 0.094 for* TLR2 *and* TLR4*, resp.) (Figures 1(c) and 1(d)). Furthermore, a positive correlation between the RQ values of* TLR2* and* TLR4* mRNA before and after treatment considering only the eradicated patients was found (before: *r* ^2^ = 0.85, *P* < 0.0001; after: *r* ^2^ = 0.55, *P* = 0.006).

The influence of* cagA *and* vacA* bacterial genotypes on the gene expression levels, both before and after treatment (Table 3), showed no evidenced significant difference between* cagA*+ and* cagA*− genotypes (*P* > 0.05) for both analyzed genes. Similarly, no significant difference was observed regarding* VacA *sm genotype. We also evaluated the association between relative expression levels of* TLR2* and* TLR4* mRNA and the risk factors such as age, gender, smoking, drinking, and histological type of gastric lesion. None of the factors investigated showed significant differences (data not shown).

### 3.2. TLR2 and TLR4 Protein and mRNA Relative Expressions Are Concordant

**Figure 2:**
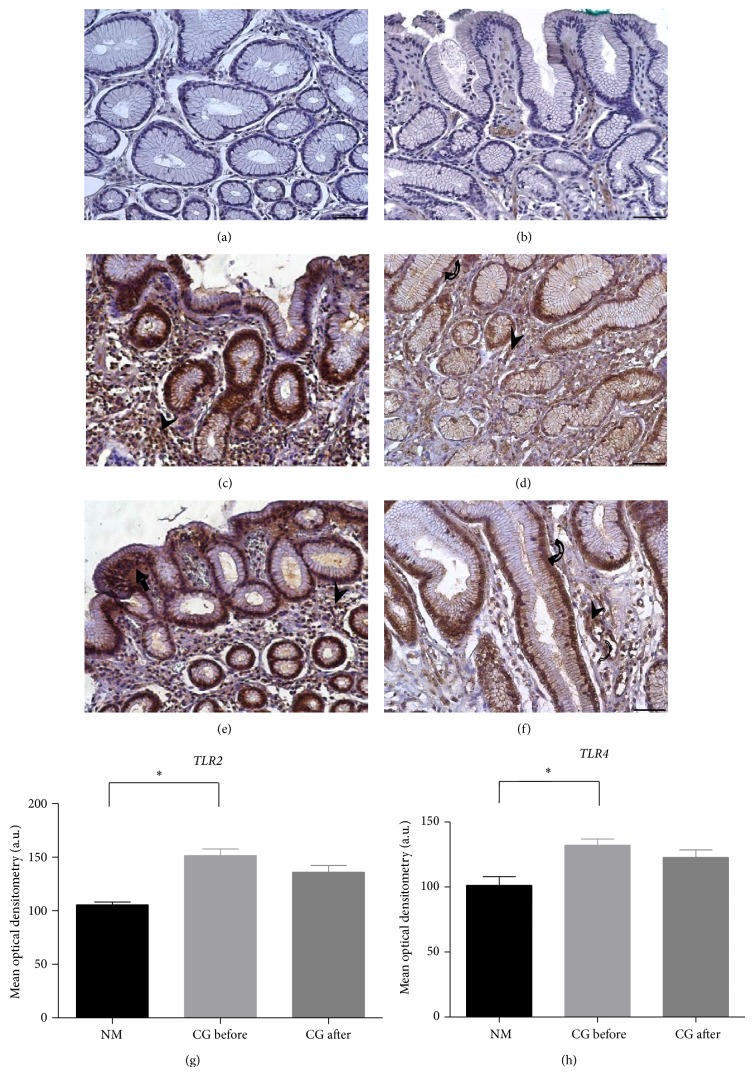
Immunohistochemistry images of Toll-like receptors (TLRs) in normal gastric mucosa (NM) and chronic gastritis (CG). Normal mucosa ((a) TLR2 and (b) TLR4); normal glands with no staining or low expression intensity;* H. pylori*-positive chronic gastritis ((c) TLR2 and (d) TLR4); foveolar epithelial cells and glands before treatment, presenting moderate to strong TLR expression compared to normal mucosa; ((e) TLR2 and (f) TLR4); foveolar epithelial cells and glands after bacteria eradication (no significant reduction of protein expression was detected). Counterstain: hematoxylin. Bars: 50 *μ*m. ((g)-(h)) Densitometry analyses (mean ± SE). ^*^ *P* < 0.05. a.u. = arbitrary unit.

In normal mucosa, the TLR2 and TLR4 protein expression was weak or absent, mainly in the foveolar epithelium (Figures 2(a) and 2(b)). Nevertheless, the CG-Hp+ samples collected before the treatment showed a cytoplasmatic, perinuclear, and focal immunostaining pattern, mostly in the basal area of the foveolar epithelium. A strong expression in the inflammatory cells was also observed (Figures 2(c) and 2(d)). After the eradication of* H. pylori*, an immunostaining pattern similar to the one observed before the treatment was found for both TLR2 and TLR4 proteins (Figures 2(e) and 2(f)).

The mean optical densitometry values observed in the normal Hp- group for TLR2 and TLR4 were 105.6 ± 2.7 and 101.4 ± 6.5, respectively. While the CG-Hp+ group before treatment presented significantly increased mean values for both TLR2 (151.7 ± 6.1) and TLR4 (132.2 ± 4.7) in comparison with the normal Hp- group (*P* = 0.020 and *P* = 0.007, resp.). After eradication of the bacteria, both TLR2 and TLR4 proteins showed a slight reduction in their mean optical densitometry values (136.1 ± 6.1 and 122.8 ± 5.8, resp.). However, there were no significant differences between these values before and after treatment (*P* = 0.064 and *P* = 0.198, resp.) (Figures 2(g) and 2(h)), confirming the findings regarding the mRNA relative expression.

## 4. Discussion 

In this study we investigated for the first time the occurrence of alterations in the* TLR2* and* TLR4* mRNA and protein expression in* H. pylori*-infected patients with chronic gastritis, before and after successful bacteria eradication treatment. Our results did not reveal significant changes in the relative expression levels of either* TLR2* or* TLR4* mRNA after treatment in eradicated patients, which was confirmed by immunohistochemistry. Moreover, the mRNA expression of both receptors remained increased after eradication therapy compared to the normal Hp- group, showing that the eradication of the bacteria did not normalize the expression of these receptors, at least under the conditions evaluated. Additionally, we also observed a positive correlation between the mRNA expression values of* TLR2* and* TLR4* confirming that* H. pylori* activates both receptors.

TLRs are transmembrane proteins that play a critical role in the recognition of pathogen components [24]. LPS of Gram-negative bacteria are recognized mainly by TLR4 and also TLR2 activating signaling pathways that culminate in an inflammatory response [25]. It is believed that the interaction between bacterial virulence and a genetically susceptible host is associated with more severe chronic inflammation, which may, in the long run, lead to cancer [26]. Under normal physiological conditions, the expression of these receptors in the mucosa of the gastrointestinal tract is low due to the action of their antagonists, such as TOLLIP (Toll-interacting protein) and PPAR*γ* (Peroxisome proliferator-activated receptor) that show higher levels in order to prevent inappropriate activation of nonpathogenic antigens [27–29].

In our study, we observed a slightly increased expression of both* TLR2* and* TLR4* in CG-Hp+ patients even after successful* H. pylori* eradication compared to the noninfected normal mucosa. In children infected with* H. pylori*, Lagunes-Servin et al. (2013) [30] found an increase in the expression of the TLR2, TLR4, TLR5, and TLR9 in the gastric epithelium compared with noninfected children and also an association with pro- and anti-inflammatory cytokines (IL-8, TNF-*α*, and IL-10). These findings confirm that* H. pylori* has the ability to increase the* in vivo* expression of TLRs by gastric epithelial cells early during infection in children, starting a chronic and balanced inflammatory process that will continue for decades, and so may contribute to the development of* H. pylori*-associated diseases later in adulthood. Pimentel-Nunes et al. (2013) [31] observed that, considering the different TLRs of normal* H. pylori*-negative mucosa, the mRNAof* TLR5* was the most expressed, followed by those of* TLR2* and* TLR4*. Furthermore, these authors found* TLR2* and* TLR4* overexpression in intestinal metaplasia, independent of the* H. pylori* status, and in the dysplasia/cancer sequence. Moreover, upregulation of* TLR2* and* TLR4* mRNA was also observed in* H. pylori*-associated normal mucosa. These results were confirmed by immunohistochemical analyses, which found an increase in protein expression in* H. pylori*-infected normal mucosa, further increasing in intestinal metaplasia and dysplasia/carcinoma. These findings suggest that progressive activation of these receptors, initially not only by* H. pylori*, but also by other PAMPs (pathogen-associated molecular patterns) or DAMPs (damage-associated molecular patterns), at later stages, may play an important role in gastric carcinogenesis and tumor progression [31].

Upregulation of* TLR4* expression responsiveness to LPS and* H. pylori* in gastric cell lines has also been reported [32, 33].* H. pylori* infection induced both* TLR4* mRNA and protein expression in AGS cells that were dependent on bacterial load and infection duration. However, the transfection of AGS cells with* TLR4* siRNA followed by the bacterial infection suppressed the expression of this receptor [32]. Moreover, LPS of* H. pylori* upregulate TLR4 expression via TLR2 signaling in MKN28 gastric cell lines by the MEK1/2-ERK1/2 MAP kinase pathway [34], leading also to an increase in cell proliferation. Conversely, previous studies [35–37] did not observe any relevant role of TLR4 in the cellular recognition of* H. pylori *in AGC cells. These controversial results may be due to differences in the lipid A structures produced by distinct* H. pylori* strains [38–40]. Therefore, the interaction of the bacteria with TLR2 should also be considered, mainly after the first contact with the gastric mucosa, triggering immunologic responses [41] such as induction of IL-8 and subsequent activation of NF-*κ*B [11].

Our study revealed no reduction of the transcript levels of* TLR2* and* TLR4* or their proteins 3 months after treatment, showing that the successful eradication of* H. pylori* does not change the expression of these receptors within a short period after the treatment. Similarly, Garza-González et al. (2008) [42] found no quantitative differences in the* TLR4 *and* TLR5 *mRNA levels either, regardless of the presence or absence of* H. pylori* in gastric epithelial cells biopsies and AGS cells, suggesting that the mRNA levels of both receptors may not be influenced by the infection process or at least not at the time points selected for analysis. However, in our study, we observed higher levels of* TLR2* and* TLR4* mRNA and of both proteins in* H. pylori*-infected mucosa compared to noninfected normal mucosa. It should however be taken into consideration that the posttreatment time elapsed until biopsy collection which may not have been sufficient for mucosal renovation and transcription level normalization. Moreover, alterations in mRNA expression levels after* H. pylori* infection eradication therapy have been demonstrated, involving genes associated with cell damage, inflammation, proliferation, apoptosis, and intestinal differentiation [43, 44].

This study did not investigate the molecular mechanisms involved in the inflammatory cascade induced by* H. pylori* infection triggered by TLR4 and TLR2. Therefore, further investigations are needed to clarify the possible involvement of signaling pathway MyD88-MAPK-NF*κ*B as well as the role of PPARs (Peroxisome proliferator-activated receptors) on inhibition of pathway regulating expression of proinflammatory genes and stress kinase pathways [31, 45, 46], which suppresses inflammation in* H. pylori* infection.

When we compared the expression levels of* TLR2* and* TLR4* mRNA with risk factors and bacterial virulence genotypes, we did not find any association. The studies that assess the effects of* cagA* and* vacA* virulence factors on the gene and protein expression are controversial. Our results evidenced that there were no quantitative differences in the mRNA levels of these receptors regardless of* cagA* and* vacA* status. Similar results were reported by Garza-González et al. (2008) [42], which demonstrated that the mRNA levels of* TLR4* and* TLR5* in gastric cells both* in vivo* and* in vitro* were not influenced by the* vacA* status, suggesting that this virulence factor may not be involved in the first steps of innate immune-recognition of* H. pylori*. Another study evidenced downregulation of TLRs 2 and 5 and upregulation of TLR9 by* H. pylori* in human neutrophils regardless of cagPAI status and the integrity of T4SS [47].

In conclusion, we report a discrete increase in* TLR2* and* TLR4* mRNA and protein expression in CG-Hp+ patients before eradication therapy and the maintaining of this expression pattern even after treatment, suggesting that these receptors remain expressed in the gastric mucosa even after eradication of the bacteria, at least for the period evaluated. Therefore, considering the higher risk of malignant progression in patients infected by* H. pylori* for a long time, further investigations are needed to clarify the changes in the expression of other genes related with the inflammatory cascade induced by bacteria, such as those encoding cytokines and malignant transformation processes as well as the signaling pathways involved.

## Figures and Tables

**Table 1:** Demographic and clinicopathological data of *H. pylori*-positive patients with chronic gastritis.

Patients	Total *N* = 59
Age	
Mean (SD), years	48.0 ± 15.9
Range	21–82
Gender	
Male	26 (44%)
Female	33 (56%)
Drinking	
Yes	19 (32%)
No	36 (61%)
Not available	4 (7%)
Smoking	
Yes	21 (36%)
No	36 (61%)
Not available	2 (3%)
Histological diagnosis	
Chronic gastritis	45 (76%)
Atrophic gastritis	8 (14%)
Atrophic gastritis-associated intestinal metaplasia	6 (10%)
Eradication therapy	
Completed treatment	37/59 (63%)
Bacteria eradication	23/37 (62%)
Bacteria noneradication	14/37 (38%)

*N*: number of individuals.

**Table 2:** Comparison of *TLR2* and *TLR4* mRNA relative expression levels before and after *H. pylori* eradication therapy.

	Before treatment	After treatment
	*N*	*N*
*TLR2 *		
Completed treatment	37	37
RQ median	1.31	1.55
Range	0.37–23.05	0.34–43.12
*P* value	0.291	
Eradicated	23	23
RQ median	1.32	1.47
Range	0.37–12.63	0.34–43.12
*P* value	0.533	
Noneradicated	14	14
RQ median	1.23	1.83
Range	0.66–23.05	0.37–6.36
*P* value	0.357	
*TLR4 *		
Completed treatment	37	37
RQ median	1.45	1.64
Range	0.50–11.09	0.56–28.07
*P* value	0.084	
Eradicated	23	23
RQ median	1.26	1.53
Range	0.50–7.17	0.64–28.07
*P* value	0.094	
Noneradicated	14	14
RQ median	1.88	1.99
Range	0.54–11.09	0.56–9.75
*P*value	0.626	

*N*: number of individuals; *P*: probability; RQ: relative quantification; statistical analysis by Wilcoxon signed rank test.

**Table 3:** Comparisons of *TLR2* and *TLR4* mRNA expression levels according to *cagA* and *vacA* genotypes of *H. pylori* in infected patients before and after bacteria eradication treatment.

	*TLR2 *			*TLR4 *		
	Samples (%)	RQ median (range)	*P* value	Samples (%)	RQ median (range)	*P* value
Before treatment						
*cagA+ *	11/23 (48.0)	1.32		11/23 (48.0)	1.75	0.689
		0.66–23.05	0.518		0.65–11.09	
*cagA− *	12/23 (52.0)	1.41		12/23 (52.0)	1.80	
		0.71–12.63			0.97–7.17	
*vacA s1m1 *	12/25 (48.0)	1.51		12/25 (48.0)	1.97	0.978
		0.67–12.63	0.849		0.65–7.17	
*vacA others *	13/25 (52.0)	1.31		13/25 (52.0)	2.02	
		0.66–23.05			0.97–11.09	
After treatment						
Eradicated						
*cagA*+	5/11 (46.0)	1.55		5/12 (42.0)	1.61	0.876
		0.50–7.21	0.662		1.06–12.01	
*cagA− *	6/11 (54.0)	2.16		7/12 (58.0)	2.69	
		0.59–43.12			0.64–28.07	
*vacA s1m1 *	7/13 (54.0)	0.71		7/13 (54.0)	1.42	0.234
		0.34–7.21	0.234		0.64–12.01	
*vacA *others	6/13 (46.0)	2.65		6/13 (46.0)	3.30	
		0.59–43.12			0.89–28.07	
Noneradicated						
*cagA+ *	6/12 (50.0)	1.87		6/10 (60.0)	1.99	0.762
		1.03–5.61	0.699		0.56–3.92	
*cagA− *	6/12 (50.0)	1.34		4/10 (40.0)	2.59	
		0.37–6.36			0.82–9.75	
*vacA s1m1 *	5/12 (42.0)	1.36		4/11 (36.4)	1.66	0.230
		0.65–5.61	0.639		0.56–2.99	
*vacA others *	7/12 (58.0)	1.92		7/11 (63.6)	3.52	
		0.37–6.36			0.82–9.75	

*vacAothers* (*s1m2, s2m2, s1_, s2_,* and _*m1*); *P* value = Mann-Whitney test; *P* < 0.05.
